# Association between type of anticoagulation and blood transfusion requirements during renal replacement therapy in the ICU

**DOI:** 10.1186/cc9545

**Published:** 2011-03-11

**Authors:** A Iyer, J Ewer, L Tovey, H Dickie, M Ostermann

**Affiliations:** 1 Guy's & St Thomas' Foundation Hospital, London, UK

## Introduction

Renal replacement therapy (RRT) is an essential component of modern critical care. Anticoagulation is necessary to prevent premature clotting of the extracorporeal circuit. We aimed to determine whether regional anticoagulation with citrate is associated with the reported reduced need for blood transfusions compared with heparin or epoprostenol.

## Methods

We retrospectively analysed all of the adult patients who received RRT in the general ICU at Guy's & St Thomas' Hospital, London between October 2008 and March 2009. Our first-line anticoagulation was heparin delivered via the circuit. It was clinical practice to maintain patients' haemoglobin (Hb) at 8 g/dl. We calculated the number of units of red blood cells (RBC) transfused during the course of RRT and for 24 hours after.

## Results

In total, 156 patients were treated with RRT during the 6-month period. One hundred and forty-two patients received a single type of anticoagulation throughout the whole course of RRT (heparin via the circuit or systemically, *n *= 85; citrate, *n *= 12; epoprostenol, *n *= 45). Among patients without overt clinical bleeding episodes, the number of RBCs needed per day of RRT to maintain Hb at 8 g/dl was 0.5 units on citrate, 0.6 units on heparin and 0.6 units on epoprostenol (*P *= NS). Among 14 patients who had clinically recognized bleeding problems and did not change their anticoagulation, the requirements for RBC transfusion were 4.8 units/day in patients on heparin, 2.8 units/day on epoprostenol and 1.7 units on citrate (*P *= NS). In 11 patients, anticoagulation was changed during the course of RRT because of bleeding problems. Of the seven patients started on heparin, three were changed to citrate and four to epoprostenol. Four patients had a change from epoprostenol to citrate. Change from heparin to citrate resulted in reduced transfusion requirements from 0.8 units RBC per RRT day to 0.6 units per day (*P *= NS). Changing from heparin to epoprostenol was associated with a reduction from 8.1 to 0.73 units RBC per day on RRT (*P *= NS).

## Conclusions

Citrate-based anticoagulation for RRT in patients with contraindications to heparin was not associated with lower transfusion requirements.

